# Supplementary material for: The interactome of intact mitochondria by cross-linking mass spectrometry provides evidence for coexisting respiratory supercomplexes
Source: Mol Cell Proteomics. 2017 Dec 8;17(2):216–32. doi: 10.1074/mcp.RA117.000470 (PMC5795388; doi:10.1074/mcp.RA117.000470)
Supplement: Supplemental Data [file supp_RA117.000470_133922_0_supp_23978_fzffwf.zip › spectra_annotation/mito_DR_spectra_annotation/302-1-4-1-8-1.pdf]

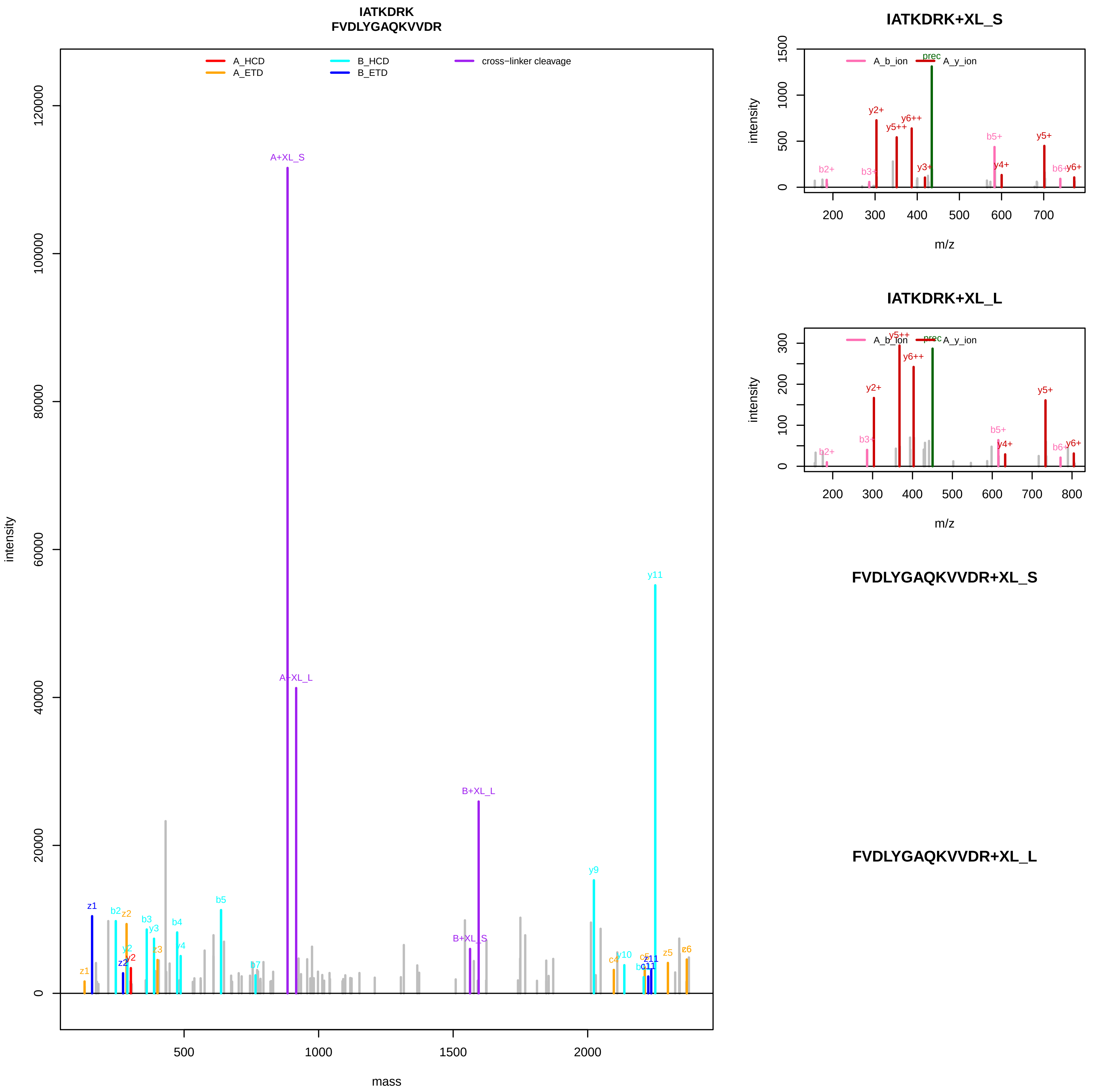
IATKDRK
FVDLYGAQKVVDR
A_HCD
A_ETD
B_HCD
B_ETD
cross−linker cleavage
A+XL_S
A+XL_L
B+XL_L
B+XL_S
y11
y9
y10
b5
b4
b3
y3
y4
b2
z1
z2
y2
z3
b7
c4
z5
z6
c11
z11
intensity
mass
IATKDRK+XL_S
IATKDRK+XL_L
A_b_ion
A_y_ion
prec
y2+
y5++
y6++
y3+
y4+
y5+
y6+
b2+
b3+
b5+
b6+
m/z
FVDLYGAQKVVDR+XL_S
FVDLYGAQKVVDR+XL_L
